# An atypical lateral hernia and concomitant inguinal and umbilical hernias in a patient with polycystic kidney disease and an intracranial aneurysm – a combined approach of clinical and radiological investigation, endoscopic hernia repair, and anatomical cadaver model documentation and a systematic review of the literature

**DOI:** 10.1186/s40064-015-0857-2

**Published:** 2015-02-14

**Authors:** László Veréb-Amolini, Thomas Betschart, Emilia Kiss, Oliver Ullrich, Stefan Wildi, Elisabeth Eppler

**Affiliations:** Department of Surgery, Waid Hospital, Tièchestrasse 99, Zürich, CH-8037 Switzerland; Department of Radiology, Waid Hospital, Tièchestrasse 99, Zürich, CH-8037 Switzerland; Institute of Anatomy, University of Zürich, Winterthurerstrasse 190, Zürich, CH-8057 Switzerland; Institute of Neuroradiology, University Hospital, Otto-von-Guericke-University, Leipziger Strasse 44, Magdeburg, D-39120 Germany; Current address: Department of Anatomy II, Friedrich Alexander University Erlangen-Nürnberg, Erlangen, D-91054 Germany

**Keywords:** Para-inguinal hernia, Lateral ventral hernia, Interparietal hernia, Spigelian hernia, Endoscopic hernia repair, Polycystic kidney disease, Intracranial aneurysm, ADPKD, Kidney transplant

## Abstract

Atypical hernias are difficult to diagnose due to their rarity and often unspecific symptoms. In the literature there exist hints to peri-inguinal hernias, i.e. direct lateral hernia, but most of them are forms of Spigelian hernias. Since the majority were described during the first half of the past century or even earlier, only very few cases have been documented using modern diagnostic techniques. We report a unique case of a 51 year old patient presenting with an atypical inguinal hernia with concomitant inguinal and umbilical hernias in combination with cystic kidney disease and intracranial aneurysm. The atypical position of the hernia was assumed from clinical inspection, ultrasound and CT scan and verified during pre-peritoneoscopy. Using an anatomical cadaver dissection approach, we followed the unusual position of the hernia through the abdominal wall below the aponeurosis of the external oblique muscle. After a thorough literature search, we assume that the present hernia containing a hernial sac has not been documented before, especially not in such a multidisciplinary approach comprising radiological, surgical and anatomical localisation and endoscopic treatment in a patient with a clinical situation being aggravated by large cystic kidneys leading to dialysis-dependency. Rare hernias have been described as being often associated with concomitant inguinal or other hernias, a predisposition for the male gender and a pathogenic mechanism related to other soft tissue defects such as cystic kidney disease or cranial aneurysm. Thus, we consider this a unique case that has not been documented in this constellation previously, which may increase the awareness for these rare hernias.

## Introduction

Atypically located hernias can present as difficult cases to diagnose, not only due to their rarity. Sometimes unclear symptoms and a vague lump or tenderness are found at clinical presentation instead of a palpable lump with the classical signs of a hernia (Light et al. 2013). Atypically located ventral hernias are often classified amongst the Spigelian hernias, which are rare hernias (1–2% of cases) of the lateral abdominal wall. Most of these hernias are located in the “Spigelian hernia belt”, a transverse 6-cm-wide zone above the interspinal plane. Spigelian hernias form a protrusion of pre-peritoneal fat, peritoneal sac, or organ(s) through a congenital or acquired defect in the Spigelian aponeurosis, i.e. the aponeurosis of the transversus abdominalis muscle delineated by the lateral edge of the rectus abdominalis muscle medially and the semilunar (Spigelian) line laterally, first described by the Belgian anatomist Adriaan van der Spieghel in 1645 (Skandalakis et al. 2006; Ghosh et al. 2014). Here we describe a very rare lateral hernia, which does not present as a typical Spigelian hernia, but is located much lower, lateral and cranial to the inner inguinal ring. Previous descriptions of similar atypical ventral hernias classified them as e.g. para-inguinal (Grierson and Leacock 1949) or peri-inguinal (Gallese 1991), however, a formal systematic classification does not exist in the literature. Thus far, there are very few case reports on rare hernias and those existing reports are mainly from the first half of the last century, which were hampered by the lack of suitable diagnostic methods (e.g. Holloway 1922). Today, modern imaging techniques, laparoscopic or pre-peritoneoscopic exploration and repair are feasible and well-established for the management of hernias, even Spigelian hernias (Moreno-Egea et al. 2002), however, data for Spigelian hernias are still very scarce due to the general rarity of this hernia type (Salameh 2008; Rath et al. 2013). To the best of our knowledge, only a single case report exists so far on the laparoscopic repair of a rare ventral hernia lateral and cranial to the inner inguinal ring (Yokoyama et al. 2013), however, adipose tissue was its sole content. Laparoscopic exploration does not always reveal the distinct localisation of an interstitial hernia. The hernia we present here was preoperatively localised by clinical inspection, ultrasound and CT scan and photo-documented during endoscopic treatment. Since pre-peritoneal exploration did not allow a tracking of the hernia through the abdominal wall, we documented the constitution of the abdominal wall at the assumed localisation of the hernia by a reconstruction of the surgical site using an anatomical cadaver model with the respective landmarks. To the best of our knowledge, this is the first atypical hernia documented by such an interdisciplinary approach with the integration of a cadaver model. This is also the first case of a pre-peritoneoscopic repair of a rare hernia type in a patient with dialysis-dependent kidney disease and intracranial aneurysm. Based on the sparsely documented cases in the literature, which we thoroughly summarise (Table 1), we propose a systematic nomenclature for these rare hernias.

**Table 1:** **Summary of the reported hernias from the literature**

**Author**	**Year**	**Patient**	**Description of intraoperativ finding**	**Classification by author**	**Classification by Gallese**		
					**Parainguinal**	**Periinguinal**	**Spigelian?**
Charles Greene Cumston	1904	9 year old girl	Origin in the internal ring but aside in abnormal position containing 3 small lipomas, plus a direct hernia	Interstitial Hernia	X		
Jackson K.Holloway	1922	45 year old woman	8 cm above and lateral to the internal ring containing appendix which was removed	Lateral ventral Hernia			X
Alexius McGlannan	1927	50 year old man	interstitial between transveralis and internal oblique muscle	Lateral ventral Hernia			X
William E.Lower, N.Fred Hicken	1931	54 year old man	Opening just above the internal inguinal ring (containing omentum), no communication with the inguinal canal, no inguinal hernia demonstrable	Parainguinal interstitial Hernia		X	
William E.Lower, N.Fred Hicken	1931	41 year old woman	Orifice just lateral and above the internal inguinal ring, Lig. Rotundum through the internal ring	Parainguinal interstitial Hernia		X	
John Grierson, Aubrey Leacock	1949	59 year old woman	Neck about one inch above the internal ring no inguinal hernia	Parainguinal Hernia		X	
John Grierson, Aubrey Leacock	1949	35 year old man	Direct hernia and 2nd orifice 1 inch above the internal ring (lliohypogastric nerve)	Parainguinal Hernia		X	
Sten Ulbak Jørgen Ørnsholt	1982	36 year old man	Internal Aperture in the inguinal canal, Fundus lateral and above the internal ring containing sigmoid loop	Parainguinal Hernia	X		
Nando Gallese	1991	maschio 55 anni	Hernia inguinale diretta, obliqua esterna, voluminoso lipoma preerniario e piccola hernia periinguinale	Hernia Periinguinale (adiposo)		X	
Nando Gallese	1991	maschio 50 anni	Hernia inguinale diretta e difetto circulare del muscoli piccolo obliquo e trasverso	Hernia Periinguinale (francia epiploica)		X	
Giuseppe Cavallaro et al.	2007	maschio 79 anni	ansa ileale al di sopra del canale inguinale	Periinguinal Hernia		X	
Takahide Yokohama et al.	2013	81 old woman	Bilateral femoral, left indirect and right direct hernia and a hernia with an orifice lateral and cranial ot the internal ring	Lateral ventral Hernia: preaperitoneal fat with concomittant (right illiac) vessel		X	
Own case	2015	51 year old man	Atypical Hernia laterally to the non enlarged inner inguinal ring and direct hernia, recurrent combined hernia (direct hernia and praeperitoneal Lipoma) on the opposite side plus umbilical hernia	Periinguinal Hernia		X	

For all references to interstitial hernia see Greene Cumston 1905, McGlannan 1927, Lower and Hicken 1931.

For Spigelian Hernia Panagiotis N. Skandalakis (overview) 2006.

For Hernia extending into the inguinal canal Greene Cumston 1905, Ulbak and Ørnsholt 1983.

All other hernias are presented by the authors themselves and fits to hernias that have no communication with the inguinal canal Lower and Hicken 1931, Grierson and Leacock 1949, Gallese 1991, Cavallaro et al. 2007, Yokoyama et al. 2013.

## Case report

### Clinical case

A 51 year old patient with dialysis-dependent cystic kidney disease was presented by the nephrology unit to the emergency unit of our hospital due to his primarily non-repositionable umbilical hernia. In his history, a bleeding aneurysm of the anterior communicating artery had been clipped, three years ago.

Clinical investigation revealed an umbilical hernia of 1 cm × 1 cm in size, which could be repositioned without difficulty. In addition, with the patient in an upright position, on the left side, a discrete tumour was detected of 3 cm × 3 cm in size with protrusion into the inguinal canal and with an enlargement of the superficial inguinal ring to 1.5 cm. Several decades ago the patient had a surgical repair of an inguinal hernia at this site. Clinically, we also found a protrusion of 7.5 cm × 7.5 cm in size on the right side, however, not presenting as typical inguinal hernia. Written informed consent for publication of this case was obtained from the patient whom the authors herewith gratefully acknowledge.

### Ultrasound

**Figure 1:**
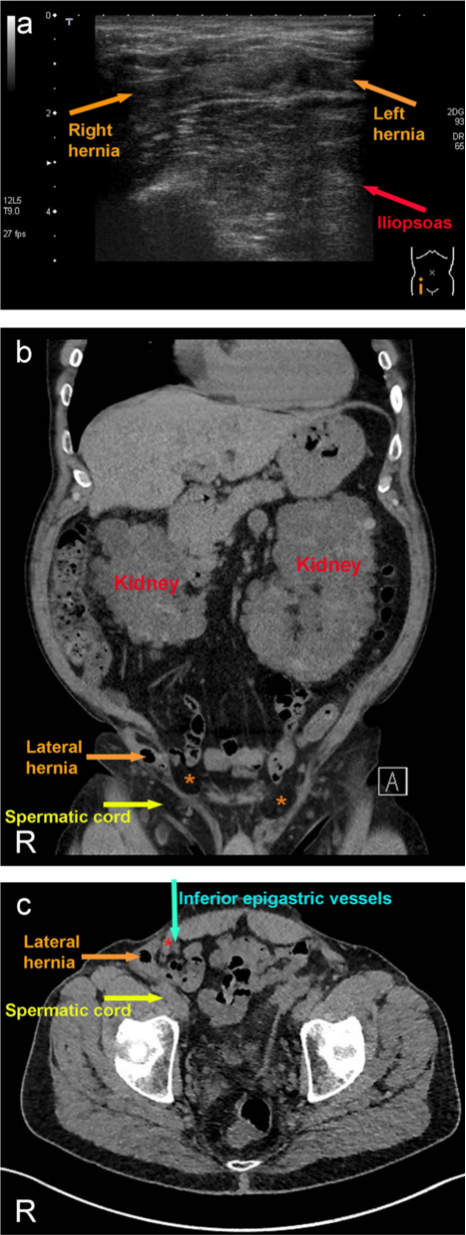
**Pre-operative localization of the hernia using (a) ultrasound and (b,c) CT. (a)** Hernia on the right side (orange arrow) movable, atypically lateral position. Red arrow points to the iliopsoas muscle. **(b)** Coronal CT scan demonstrates direct inguinal hernias on both sides (orange asterisks) within the transversal fascia as well as the very lateral inguinal hernia with adipose tissue and small intestine (orange arrow) lateral to the spermatic cord (yellow arrow) on the right side. Note enlarged polycystic kidneys. **(c)** Horizontal CT scan reveals the position of the lateral hernia (orange arrow) latero-dorsal to lateral abdominal wall muscles (red asterisk), in particular internal oblique and transversus abdominalis muscles, to the inferior epigastric vessels (turquoise arrow), and the inguinal ligament, and lateral to the spermatic cord (yellow arrow).

Abdominal ultrasound (linear array sonde, 7 MHz) confirmed the umbilical hernia containing adipose tissue with a hernial sac of 1.2 cm. Nearby on the left side, an inguinal hernia was visible with a hernial sac of 2.5 cm containing adipose tissue. On the right side, another inguinal hernia was visible with a hernial sac of 0.9 cm containing adipose tissue. In addition, on the right side, a movable inguinal hernia in an atypically lateralised position was suspected (Figure 1a).

### CT scan

Native CT scan (multislice-helical CT, 0.6 mm collimation, axial and coronal reconstructions, 2 mm slice thickness) confirmed a hernial sac containing adipose tissue and small intestine without signs of complications. The hernial sac was localised directly lateral-dorsal to the oblique and transversus abdominalis muscles and the inguinal ligament and cranial to the spermatic cord. The hernial sac protruded in a latero-cranial direction below the aponeurosis of the external oblique muscle. Distinct to this, indirect lipoma (left) and direct hernias were discriminated coated by transversal fascia. As a secondary finding, enlarged polycystic kidneys of 25 cm and 27 cm, respectively, were visible (Figure 1b,c).

### Endoscopic hernioplasty and postoperative follow-up

**Figure 2:**
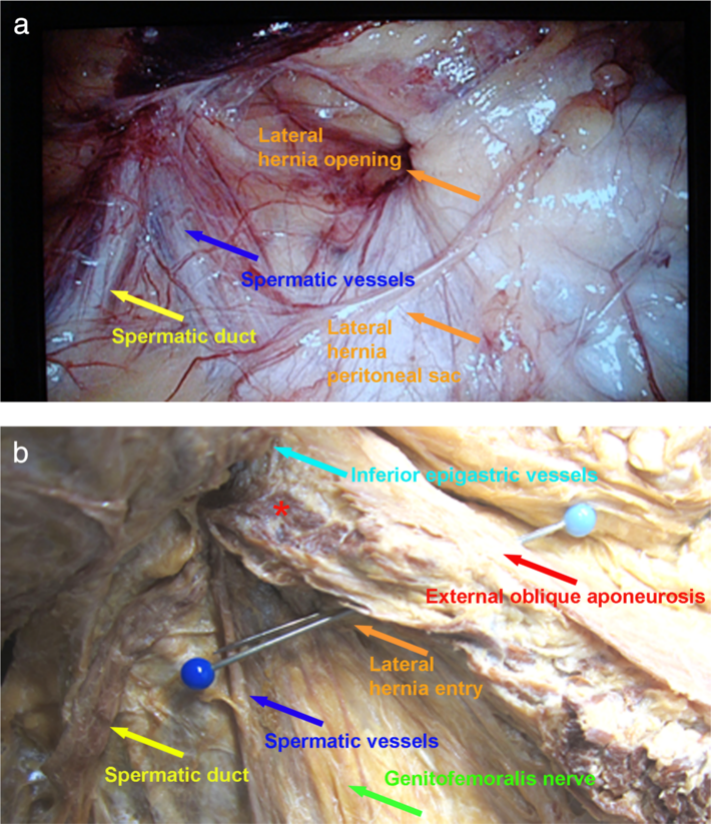
**Endoscopic (a) and anatomical cadaver (b) site documentation. (a)** Lateral ventral hernia (orange arrows) lateral to the spermatic duct (yellow arrow) and the spermatic vessels (blue arrow) uniting at the inner inguinal ring. **(b)** The needle marks the hernia position between the sheaths of the lateral abdominal wall muscles. Hernia entry into the lateral abdominal wall is indicated by an orange arrow. The hernia remains within the abdominal wall (red asterisk designates transversus abdominalis muscle and internal oblique muscle fibres) below the aponeurosis of the external oblique abdominalis muscle (red arrow). The hernia is situated lateral to the spermatic duct (yellow arrow) and spermatic vessels (blue arrow) used as landmarks for the cadaver reconstruction of the hernia position. Note inferior epigastric vessels (turquoise arrow) and the genitofemoralis nerve (green arrow).

Endoscopic pre-peritoneal hernioplasty was performed without complications, whereby on the left side we found scar tissue from previously described surgical interventions. Furthermore, as expected we found a direct hernia and a pre-peritoneal lipoma, which could be repositioned. On the right side, a hernia medial to the inferior epigastric vessels was found corresponding to the expected direct hernia, but in addition, another hernial opening was detected laterally to the not enlarged inner inguinal ring containing a peritoneal hernia sac and a pre-peritoneal lipoma (Figure 2a), which both could be retracted without complications. Consecutively, on each side a 10 cm × 15 cm Parietex® mesh was pre-peritoneally positioned without slits and without need for further fixation. Subsequently, the small umbilical hernia was surgically corrected via direct suture. On first day after surgery, the patient underwent regular dialysis treatment. On the third postoperative day, the patient was discharged from hospital and regularly presented for dialysis without signs of postoperative complications. Meanwhile, during the 19 month follow-up period, successful kidney transplantation was performed about one year after hernia repair with placement of the transplant into the right iliac region followed by a further 7 months without signs of local complications.

### Anatomical reconstruction

In order to track the interstitial localisation of the hernia, we reconstructed the intraoperative situation by a cadaver dissection (Figure 2b) in a routinely formalin-fixed male corpse obtained from the institutional body donation programme (http://www.anatom.uzh.ch/Bodydonation.html) using anatomical landmarks, e.g. according to Netter (2010). The institutional body donation program follows the ethical guidelines “On the use of cadavers and parts of cadavers in medical research and for pre-, postgrad and continued education” (2008) and “Research with human subjects” (2010) by the Swiss Academy of Medical Sciences. The study was conducted in the year 2013.

### Literature search

A literature search using PubMed was performed for the key words parainguinal hernia, lateral ventral hernia, Spigelian hernia, Spieghel hernia and interstitial hernia complemented by a search for polycystic kidney disease, ADPKD, cranial aneurysm and hernia. We further searched the reference lists of defined articles for further publications. Additionally, hernia surgery and anatomy books were compared for the respective landmarks. The cases most similar to our case were collected and reviewed for a systematic nomenclature.

## Discussion

The present case is of clinical relevance for several reasons, namely that the question arose

1. whether endoscopic treatment would be possible with respect to the large cystic kidneys,
2. whether the diverse and atypical hernias could be treated endoscopically
3. what would be the distinct origin and localisation of the atypical hernia, in particular the inner hernia entry point.

Endoscopic treatment was successfully performed in a single session. Since the atypical hernia does not fit into the common pattern of inguinal hernias, we at first considered whether this was a so-called low Spigelian hernia. As described above, the respective hernias were situated along the semilunar line defined as the lateral edge of the rectus abdominalis muscle. Thus, Spigelian hernias are located laterally to the rectus abdominalis muscle aponeurosis and, in particular, below the arcuate (semi-circular) line of Douglas, mainly within the “spigelian hernia belt”, a transverse 6-cm-wide zone above the interspinal plane (Skandalakis et al. 2006). Typically, they are localised above the triangle of Hesselbach, whereby the so-called low Spigelian hernias are sometimes also located within that triangle. Thus, discrimination between these Spigelian hernias and direct hernias is not possible. Peri-inguinal and para-inguinal hernias are classified as lateral ventral hernias (Gallese 1991), which need to be discriminated from Spigelian hernias as well as from direct medial hernias. According to the definition by Gallese (1991), both peri-inguinal and para-inguinal hernias originate directly adjacent to the inguinal canal, however, below the semilunar line and remain below the aponeurosis of the external oblique muscle. Whereby, the para-inguinal hernias penetrate into the inguinal canal, while the peri-inguinal hernias cannot be followed into the inguinal canal.

In our case, the hernia was located laterally to the inferior epigastric vessels, even lateral to the inner inguinal ring, and ended in the abdominal wall above the inguinal ligament. Thus it can be classified among the lateral abdominal wall hernias according to the actual classification for primary abdominal wall hernias, where 2 types of midline hernias (epigastric and umbilical) and 2 types of lateral hernias (Spigelian and lumbar) are defined (Muysoms et al. 2009). According to this classification, we classify the present hernia among the L3 hernias with a size smaller than 4 cm as W1 hernia.

According to La Chausse (1756), a ventral hernia includes any hernia except a femoral, inguinal or umbilical, amongst which he also included para-inguinal, medial inguinal, supravesical and one reported hernia at the semilunar line (for references see: Skandalakis et al. 2006). Molliere (1877) stated that ventral hernias nearly always occur in the linea alba or just outside it or in the semilunar line of Spiegel, and on a level with a line from the anterior superior iliac spine to the umbilicus as outlined by Holloway (1922). He suggested to define a spontaneous ventral hernia as one that appears at an abnormal opening in the abdominal wall, apparently without explicable reason, but usually presenting in or near the linea alba, or the semilunar line of Spiegel. Whether our present case corresponds to the lateral abdominal hernia of Graser (1913) who defined a ventral hernia outside the Spigelian line as a “lateraler Bauchbruch” and attributed their pathogenesis to defects in muscle constitution (for reference see: Holloway 1922), is not easy to define due to the lack of radiological imaging in that work. Among the several hernias collected by Holloway (1922), a hernia described by Steimker (1912) in a male with several concomitant hernias might best correspond to our finding. Steimker’s hernia was observed 6 cm horizontally and medially from the left anterior superior spine with a deep hernial sac protruding into the muscles of the abdominal wall.

Another description was suggested by Grierson and Leacock (1949), who presented 2 cases of para-inguinal hernias whereby they defined them as originating near the inguinal canal, but without entering into it. In both cases, the origin was 1 inch, i.e. approximately 2.5 cm, above the inner inguinal ring with the hernial sac penetrating below the aponeurosis of the external oblique muscle towards the superior anterior iliac spine. A para-inguinal hernia was described by Ulbak and Ørnsholt (1983) as an atypical Spiegelian variant with preoperative radiological verification of a herniation into the abdominal wall using a barium enema. Intraoperatively, a hernia was found with the fundus in a similar position to the present one, namely above and lateral to the internal inguinal ring, but a loop of the spermatic cord was found to adhere to the hernial sac, which penetrated the abdominal wall through the internal aperture of the inguinal canal, which is clearly different from our case. Among the 2 cases reported by Lower and Hicken (1931), one hernia lying between the 2 oblique muscles was found in direct apposition to the lateral walls of the inguinal canal, piercing the internal oblique and transversus abdominalis muscles and the transversal fascia and opening into the peritoneal cavity by its own orifice lateral to and above the inner inguinal ring. According to the nomenclature by Kröhnlein, this would be termed a para-inguinal interstitial hernia to be differentiated from pro-peritoneal and superficial hernia (for references see: Lower and Hicken 1931). Cavallaro et al. (2007) reported another hernia superior to the inguinal region in a male patient that penetrated between the transversus abdominalis and internal oblique muscles. The preferred presence of interstitial hernias of the interparietal type in males and concomitant with other hernias is in agreement with early descriptions published in 1905 by Greene Cumston in the discussion of potential developmental predispositions.

What was already suspected at that time has been confirmed in the meantime. Indeed, congenital malformations such as cystic kidneys and intracranial aneurysms have been found to be more frequently present in patients with hernias, and defects of the extracellular matrix have been recognized to be involved in the biology of hernia formation (Morris-Stiff et al. 1997; Lynen Jansen et al. 2004).

Significantly greater numbers of inguinal, incisional, and paraumbilical hernias were found in autosomal dominant polycystic kidney disease ADPKD (Morris-Stiff et al. 1997). Our present case is further characterised by a history of bleeding intracranial aneurysm and kidney transplantation. Asymptomatic intracranial aneurysms were found by screening in 8% of ADPKD patients, a prevalence two to three times higher than that in the general population (Pirson et al. 2002; for further reading: Kanaan et al. 2014). Vascular abnormalities in ADPKD such as intracranial aneurysms are probably linked to mutations in PKD1 or PKD2 (Bichet et al. 2006), an integral membrane protein involved in cell-cell and cell-matrix interactions.

## Conclusions

The so-called lateral ventral inguinal hernias are not clearly differentiated so far due to their high variability and rarity. Furthermore, they are very sparsely documented, not alone because most of the cases described in the literature date from the first half of the last century. Thus, para-inguinal and peri-inguinal hernias are not clearly defined and mainly lack a clear radiological and photographical documentation. From the case presented here with a combined radiological, surgical/endoscopic and anatomical approach, we propose to term the peri-inguinal hernias as lateral direct hernias including also intraparietal, but not Spigelian hernias. Only via an endoscopic approach can the inner origin of the hernial sac be distinctly defined and only modern imaging methods can fully reveal the entire dimension of the hernia. Even combined modern radiological and surgical techniques were not sufficient to fully unravel the nature of the hernia, while anatomical reconstruction of the endoscopic site finally confirmed its interstitial situation. Extensive literature research that went beyond the key words in PubMed, but referred to older literature, finally clarified that these rare hernias were a feared issue among surgeons and were associated with a high mortality rate since they were difficult to predict without diagnostic imaging and enigmatic due to the lack of a systematic definition and their rarity. From this, we feel that the older literature as well as clinical hints such as congenital malformations, male gender and concomitant hernias at more conventional sites might be helpful for a timely diagnosis of this rare entity (see: Learning points).

### Learning points

- Preoperatively difficult to localize, by endoscopy also difficult since not all abdominal wall sheaths are separate and only viewed from “inward”.
- Often several hernias are observed in patients with an interstitial hernia.
- The vast majority are male patients.
- Congenital malformations may pre-dispose to interstitial hernias due to obstruction of the inguinal canal.
- The presence of cystic kidneys and other malformations such as intracranial aneurysms may point to inguinal malformations due to extracellular matrix deficiencies.
- Endoscopic treatment of this type of hernia is a suitable method since this hernia can also be easily closed with a mesh without modification of the method, in our case even in a patient with dialysis-dependent renal failure due to cystic kidney disease.
